# Relating latent class membership to external variables: An overview

**DOI:** 10.1111/bmsp.12227

**Published:** 2020-11-16

**Authors:** Zsuzsa Bakk, Jouni Kuha

**Affiliations:** ^1^ Department of Methodology and Statistics Leiden University The Netherlands; ^2^ Department of Statistics London School of Economics and Political Science London UK

**Keywords:** latent class analysis, covariates, distal outcome, three‐step estimation, two‐step estimation

## Abstract

In this article we provide an overview of existing approaches for relating latent class membership to external variables of interest. We extend on the work of Nylund‐Gibson et al. (*Structural Equation Modeling: A Multidisciplinary Journal*, 2019, 26, 967), who summarize models with distal outcomes by providing an overview of most recommended modeling options for models with covariates and larger models with multiple latent variables as well. We exemplify the modeling approaches using data from the General Social Survey for a model with a distal outcome where underlying model assumptions are violated, and a model with multiple latent variables. We discuss software availability and provide example syntax for the real data examples in Latent GOLD.

## 1 Introduction

Latent class (LC) analysis is a widely used approach in psychology and related fields for creating a grouping when the groups are unknown, based on a set of observed indicator variables. Examples include clusters of juvenile offender types (Mulder, Vermunt, Brand, Bullens, & Van Merle, 2012), students' strategy choices in solving mathematical problems (Fagginger Auer, Hickendorff, Van Putten, Bèguin, & Heiser, 2016), types of learning disabilities (Geary et al., 2009), clusters of tolerance for nonconformity (McCutcheon, 1985), and partitioning of new political parties in volatile systems (Mustillo, 2009).

In most applications, establishing such an LC measurement model and describing the distribution of the respondents across the classes is just the first step of the analysis. The interest of researchers lies also in relating this clustering to its antecedents and consequences in more complex, structural models. For example, Mulder et al (2012) related the juvenile offender profiles to more than 80 outcomes, such as recidivism. This is known as LC modelling with distal outcomes. Alternatively, predictors (covariates) of LC membership can be used to explain the clustering. For example, Mccutcheon (1985) latent examined the associations of education and age with Americans' patterns of tolerance towards nonconformity.

Until recently there were two main approaches to relating LC membership to external variables, the so‐called one‐step and (naive) three‐step approaches. Both of them can be problematic, in different ways. The two approaches differ in whether or not the structural and measurement models are estimated simultaneously. In one‐step modelling they are, in which case the two models can influence each other in ways which distorts the estimated structural model (Nylund‐Gibson, Grimm, & Masyn, 2019; Petras & Masyn, 2010; Vermunt, 2010). The naive three‐step method, in contrast, estimates the measurement model separately, but this too can produce biased estimates of the structural model, now as a consequence of ignoring the classification error that is introduced in the second (classification) step of this method.

To overcome these challenges, alternative approaches have been developed in the last decade. The two main developments are bias‐adjusted three‐step approaches and the two‐step approach. All of them are ‘stepwise’ procedures which start by estimating the measurement model alone, in the same way as in the naive three‐step method, but they then proceed in different ways to avoid its biases.

While many of these new approaches are promising, for applied researchers it is difficult to choose which one to use, as developments in the field are fast and simple guidelines are scarce. A recent overview focusing on distal outcome models is given by Nylund‐Gibson et al. (2019), but no clear guidelines are available for other situations, such as models with covariates, larger structural models which combine covariates and distal outcomes, models with multiple latent variables, or models which include direct effects of covariates on the indicators. In this paper we provide an overview of the existing approaches for relating LC membership to external variables, and provide practical guidance on the choice of modelling approach under these different circumstances.

Some of these methods have also been extended to still more complex models, such as time‐to‐event distal outcomes (Lythgoe, Garcia‐Fiñana, & Cox, 2019), latent Markov models, and latent growth models (Di Mari & Bakk, 2018; Fagginger Auer et al., 2016). Research into these extensions is developing and still scarce, and they will not be included in this overview.

In the rest of this paper, we first introduce the basic LC model and the different methods of estimating structural models, discussing their definition, properties, and implementation in existing software. We then provide an overview of the advantages and disadvantages of these different modelling approaches, focusing first on the simple situation with a single distal outcome or a covariate, and then commenting on more complex models. We then give some illustrative examples and a concluding discussion.

## 2 Latent class model with external variables, and methods of estimation for it

### 2.1 Definition of the model

Consider the vector of responses $Y_{i}=\left(Y_{i1},\ldots ,Y_{\mathrm{iK}}\right)$, where $Y_{\mathrm{ik}}$ denotes the response of individual i on one of K categorical indicator variables, with 1≤k≤K and 1≤i≤N. Latent class analysis assumes that respondents belong to one of the T categories (‘latent classes’) of an underlying categorical latent variable X which affects the responses (Goodman, 1974; Hagenaars, 1990; McCutcheon, 1987). The model for **Y** *_i_* can then be written as

(1) $$p\left(Y_{i}\right)=\sum_{t=1}^{T}p\left(X=t\right)p\left(Y_{i}|X=t\right),$$

where p(X=t) is the (unconditional) probability of belonging to latent class t, and $p(Y_{i}|X=t)$ the class‐specific probability of a pattern of responses to the indicators (throughout this paper, p(·) and p(·|·) denote marginal or conditional probabilities or density functions of variables). Models for these two kinds of probabilities are known as the *structural model* and the *measurement model* of the LC model, respectively. For the measurement model, we make the ‘local independence’ assumption that the K indicator variables are independent within the latent classes, leading to

(2) $$p\left(Y_{i}\right)=\sum_{t=1}^{T}p\left(X=t\right)\prod_{k=1}^{K}p\left(Y_{\mathrm{ik}}|X=t\right).$$

**Figure 1:**
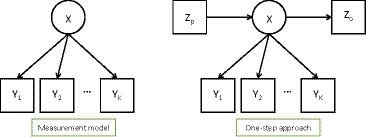
The basic latent class model (measurement model) and the one‐step model including covariates $Z_{p}$ and distal outcomes $Z_{o}$.

We refer to this as the *basic* LC model. It is represented by Figure 1(left). The number of classes T is selected by comparing the goodness of fit of models with different values of T using model selection tools such as the Akaike information criterion and the Bayesian information criterion, The entropy of the model (see Magidson, 1981), which indicates how well the class membership can be predicted by the observed variables, can be used as an additional tool to evaluate the LC solution. As this statistic focuses on the estimated measurement model, it is best used with a model like (2) without external covariates or outcomes.

The extensions of the basic LC model (2) which concern us here are ones where the model also includes observed predictors (covariates) of the latent class variable X, denoted here by the vector $Z_{\mathrm{pi}}$, and/or distal outcomes of it, denoted by $Z_{\mathrm{oi}}$. With them, the model is of the form

(3) $$p\left(Y_{i},Z_{\mathrm{oi}}|Z_{\mathrm{pi}}\right)=\sum_{t=1}^{T}\left[p\left(Z_{\mathrm{oi}},X=t|Z_{\mathrm{pi}}\right)\prod_{k=1}^{K}p\left(Y_{\mathrm{ik}}|X=t\right)\right]$$

where

(4) $$p\left(Z_{\mathrm{oi}},X=t|Z_{\mathrm{pi}}\right)=p\left(X=t|Z_{\mathrm{pi}}\right)\;p\left(Z_{\mathrm{oi}}|X=t,Z_{\mathrm{pi}}\right).$$

This is represented by Figure 1(right). In many applications the model of interest includes only covariates $Z_{\mathrm{pi}}$ or only distal outcomes $Z_{\mathrm{oi}}$, so only one of the two models on the right‐hand side of (4) is present. On the other hand, the model could also be further extended in a number of ways. First, the structural model could consist of a longer chain of such elements, with multiple latent class variables X, multiple outcomes, and/or some variables which were covariates in some models and outcomes in others. Second, we could relax the assumption that the indicators $Y_{\mathrm{ik}}$ are conditionally independent of $\left(Z_{\mathrm{pi}},Z_{\mathrm{oi}}\right)$ given X, by letting them depend also on **Z** *_pi_* to allow for non‐invariance of the measurement model (differential item functioning), or (less commonly) letting $Z_{\mathrm{oi}}$ depend directly on **Y** *_i_*. These extensions are omitted from (4) and our main discussion for simplicity, but they will also be considered later in the paper. We will also from now on omit the respondent subscript i, with the understanding that all the expressions below are for a single respondent i.

### 2.2 The one‐step approach

‘One‐step’ estimation means simply that the full model (3) – or whatever still larger model is considered – is fitted at once, estimating both its structural and measurement models together. The estimates and their standard errors are obtained using standard maximum likelihood (ML) estimation, where (3) is the contribution of respondent i to the full likelihood. This can be done using mainstream software packages for LC analysis, in particular Latent GOLD (Vermunt & Magidson, 2005, 2016) and Mplus (Muthén & Muthén, 2017); these packages can also be used for the various stepwise methods, as discussed below.

**Figure 2:**
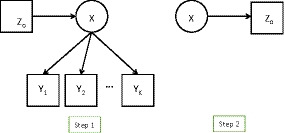
The LTB approach.

We further mention here one specialized version of one‐step estimation. This is the classify–analyse method of Lanza, Tan, and Bray (2013), here referred to as the LTB approach. It was specifically developed for distal outcome models with a continuous $Z_{o}$. The approach is based on re‐expressing the model as

$$p\left(Y,Z_{o}\right)=\sum_{t=1}^{T}\left[p\left(Z_{o},X=t\right)\prod_{k=1}^{K}p\left(Y_{k}|X=t\right)\right].$$

(5) $$=p\left(Z_{o}\right)\sum_{t=1}^{T}\left[p\left(X=t|Z_{o}\right)\prod_{k=1}^{K}p\left(Y_{\mathrm{ik}}|X=t\right)\right],$$

where $p(Z_{o})$ denotes the marginal distribution of $Z_{o}$. The rest of the last expression in (5) is an LC model where $Z_{o}$ is for the moment treated as a covariate rather than an outcome of X. This has the advantage that it avoids the need to make distributional assumptions about $Z_{o}$, misspecification of which can bias usual model estimates. The conditional distribution of X given $Z_{o}$ can be specified as a multinomial logistic model. Having fitted the model as (5), we can then reverse the conditional distributions to estimate the class‐specific conditional means of $Z_{o}$ as

$$\hat{E}\left(Z_{o}|X=t\right)=\int Z_{o}\hat{p}\left(Z_{o}|X=t\right)dZ_{o}=\int Z_{o}\hat{p}\left(Z_{o}\right)\hat{p}\left(X=t|Z_{o}\right)/\hat{p}\left(X=t\right)dZ_{o},$$

where $\hat{p}\left(Z_{o}\right)$ is the empirical distribution of $Z_{o}$ in the sample, so the integral becomes a sum over the observed values of $Z_{o}$ (see Lanza et al., 2013). This approach is represented by Figure 2. It can also be used when $Z_{o}$ is categorical, although its robustness advantage is then less apparent.

While Lanza et al. (2013) did not propose standard error estimators, Asparouhov and Muthèn (2014a) and Asparouhov and Muthèn (2014b)suggested using the delta method for categorical distal outcomes and so‐called approximate standard errors for continuous ones (defined as the square root of the within‐class variance divided by the class‐specific sample size). This approach is currently implemented in MPlus. In Latent GOLD bootstrap or robust standard errors can be chosen using the default settings.

### 2.3 Three‐step approaches

In any ‘three‐step’ approach, the estimation procedure is broken down into the following steps:

1. Estimate the measurement model using the basic latent class model, without external variables.
2. Assign respondents to predicted latent classes.
3. Estimate the structural models of interest for the latent classes and external variables, using the assigned classes in place of the latent classes.

Different three‐step methods differ in step 3, where bias‐corrected methods allow for the misclassification error introduced in step 2, but the classical (or ‘naive’) three‐step method does not.

Step 1 consists of fitting the basic LC model (2) with the selected number of latent classes. The assignment in step 2 is then based on

(6) $$p\left(X=t|Y=y\right)=\frac{p(X=t)p(Y=y|X=t)}{p(Y=y)}=\frac{P(X=t)P(Y=y|X=t)}{\sum_{u}p\left(X=u\right)p\left(Y=y|X=u\right)},$$

that is, the posterior probabilities that a respondent belongs to each class t given the respondent's observed response vector y, derived from the model estimated in step 1. The most common way of using these probabilities is *modal assignment*, which allocates each respondent to the class for which they have the highest posterior probability. Let us denote this assigned class by W.

**Figure 3:**
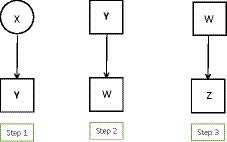
The ‘classical’ three‐step approach.

In step 3, the assigned class membership W is used in the role of X in estimating the structural model. In the classical method, this is done without any further adjustment. For example, the model (3)–(4) is simply replaced by

(7) $$p\left(Z_{o},W|Z_{p}\right)=p\left(W|Z_{p}\right)\;p\left(Z_{o}|W,Z_{p}\right),$$

where $p(W|Z_{p})$ and $p(Z_{o}|W_{i},Z_{p})$ are models of the same form as $p(X|Z_{p})$ and $p(Z_{o}|X,Z_{p})$ respectively, for example a multinomial logistic model for W and a linear regression model for a continuous $Z_{o}$. This classical three‐step approach is depicted in Figure 3.

The problem with this naive approach is that W is not necessarily equal to the true X. The assignment in step 2 thus introduces a misclassification error which can severely bias the step 3 estimates. The overall misclassification probabilities are given by

(8) $$p\left(W=s|X=t\right)=\frac{\sum_{y}p\left(Y=y\right)p\left(X=t|Y=y\right)p\left(W=s|Y=y\right)}{p(X=t)}$$

for all s,t, where each p(W=s|Y=y) is either 0 or 1 when modal assignment is used. The sum in (8) is over all the response patterns Y=y, but it is often convenient and sufficient to estimate p(Y=y) by its empirical distribution, in which case the sum will be only over those y which appear in the observed data.

*Bias‐adjusted three step approaches* were developed in order to correct for this classification error (for their details and derivations, see Asparouhov & Muthèn, 2014a, 2014b; Bakk, Tekle, & Vermunt, 2013; Bolck, Croon, & Hagenaars, 2004; Vermunt, 2010). They are based on the equality

(9) $$p\left(Z_{o},W|Z_{p}\right)=\sum_{t}p\left(Z_{o},X=t|Z_{p}\right)p\left(W|X=t\right),$$

where the empirical $P(Z_{o},W|Z_{p})$ is used to estimate $P(Z_{o},X=t|Z_{p})$, and p(W|X=t) are known from steps 1 and 2. There are two main methods, which do this slightly differently. The Bolck–Croon–Hagenaars (BCH) approach estimates the model of interest by fitting $p(Z_{o},W|Z_{p})$, but using estimation weights which are obtained from the inverse of the matrix of the misclassification probabilities p(W|X) (Vermunt, 2010). This approach is obtained, in effect, by solving (9) for the model of interest $p(Z_{o},X=t|Z_{p})$. Because of this weighting procedure some of the weights can be negative. This is a normal consequence of the weighting, and it is only a problem when it also leads to inadmissible estimates of the model parameters (which can happen especially in situations where the entropy of the measurement model is low). The approach as originally proposed by Bolck et al. (2004) did not use a weighted analysis, but rather transformed P(Z,W) into P(Z,X) using a matrix manipulation, but that implementation was limited to categorical covariates only.

For the ML approach (Vermunt, 2010), by contrast, step 3 consists of fitting the right‐hand side of (9) as a latent class model, but with p(W|X=t) treated as known numbers. This is very similar in spirit to the two‐step estimation discussed below, except that there p(Y|X=t) will be used directly, instead of p(W|X=t) here.

For both the ML and BCH approaches, correct standard errors of the estimates from step 3 need to be adjusted also for the uncertainty in the step‐two estimates (Bakk, Oberski, & Vermunt, 2014). This can be done using the general ideas of pseudo‐ML estimation introduced by Gong and Samaniego (1981). This standard error correction makes only a minor difference in most instances where the measurement model is strong, but it can be important when the measurement model is weak. It is not implemented by default in standard software, so it has to be done manually in R or Python, for example. In Latent GOLD it is possible to easily obtain the step 1 covariance matrix that is needed for the calculations; for details readers can consult the Latent GOLD upgrade manual for Latent GOLD 5.1, section 5.15 (Vermunt & Magidson, 2016). Even more importantly, when proportional assignment is used, standard errors need to be corrected for the multiple weighting, for the fact that each observation appears T times in the data for the step‐3 model. This correction can be done in standard software by using complex sampling weighting (Wedel, ter Hofstede, & Steenkamp, 1998); for details, see Bakk et al. (2014).

**Figure 4:**
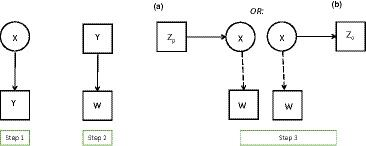
The bias‐adjusted three‐step approaches.

Bias‐adjusted three‐step methods approach LC modelling with external variables by framing it as a problem of misclassification. A different incomplete‐data formulation of the problem would be to treat it as one of missing data, where the true values of the latent class variable X are missing. One general method for dealing with such problems is multiple imputation of missing values. The simplest way of applying it here is to draw multiply imputed values of X from their posterior distribution given Y, that is, with the probabilities given by (6). This approach is known as ‘multiple pseudo‐class draws’. However, it works no better than single modal assignment of X, and multiple pseudo‐draws are indeed best seen as a variant of naive three‐step estimation. The reason for its failure is that, in order to avoid biased estimates of subsequent models of interest, multiple imputation of any missing data should be conditional on all the variables in those models of interest. Here this means that the probabilities of the draws of X should be conditional also on the external variables $\left(Z_{p},Z_{o}\right)$ and obtained from a step 1 model which includes them (where $Z_{o}$ can be included among the covariates for simplicity). This approach of *inclusive LC analysis* was proposed by Bray, Lanza, and Tan (2015), with a focus on models with distal outcomes $Z_{o}$. They showed that it can work well in this context. However, since this method includes all of the variables in its first step, it is best seen as a variant of one‐step estimation, and shares its weaknesses as well as its strengths. As such, multiple imputation from inclusive LC modelling is not discussed in more detail here (Figure 4).

### 2.4 The two‐step approach

**Figure 5:**
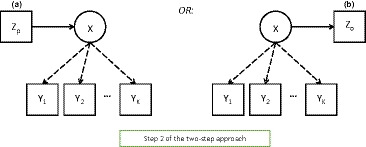
The second step of the two step approaches: the model including a covariate and the model including a distal outcome.

The ‘two‐step’ approach has the same first step as the three‐step methods, that of estimating the measurement model from the basic LC model. Then, instead of calculating any assigned latent classes W, in its second and last step it fixes the *parameters* of the measurement model at their estimated values. In other words, in step 1 we estimate model (2), and in step 2 we estimate the model of interest using (3)–(4), but with $p(Y_{\mathrm{ik}}|X=t)$ treated as known numbers rather than estimable parameters, and fixed at their estimates from step 1. This procedure is represented by Figure 5. It can be implemented with any software which would also allow one‐step estimation of the model.

The two‐step approach was first proposed for latent class models by Bandeen‐Roche, Miglioretti, Zeger, and Rathouz (1997) and Xue and Bandeen‐Roche (2002), and further developed and described by Bakk and Kuha (2018). The theory of the method can be derived from the more general idea of pseudo‐ML estimation in Gong and Samaniego (1981). The simplest way to estimate standard errors of the estimated parameters of the structural model is to simply use their standard errors from step 2 of the estimation. However, as was the case also in three‐step estimation, this ignores the uncertainty in the measurement parameters from step 1, which means that the standard errors will be underestimated to some extent. The step 1 uncertainty can also be accounted for, as described in Bakk and Kuha (2018). This makes most difference in situations where the entropy of the measurement model is low, that is, when the estimated latent classes are poorly distinguished from each other and/or the sample size is small.

## 3 Latent class modelling with external variables: recommended approaches

In this section we offer comparisons and recommendations between the methods described above, focusing on the one‐step method (including its LTB variant), the two‐step method, and the bias‐corrected (BCH and ML) three‐step methods (the naive three‐step method has essentially the same properties in almost all situations: it is straightforward to apply, but suffers from misclassification bias unless the measurement model is very strong). We consider in particular the flexibility, practicality and robustness of the methods in different situations. We discuss first models which include only external covariates or only distal outcomes, and then consider more complex models. A summary of the main conclusions from the comparisons is given in Table 1.

**Table 1:** Recommended modelling approaches for different circumstances

Approach	Distal outcome	Covariate	Large models
One‐step approach	Not recommended	Recommended with reservations	Recommended with reservations
ML	Recommended only if underlying model assumptions are met for models with continuous and count outcomes. Suitable for categorical outcomes	Recommended	Extendable to models with multiple latent variables, multiple outcomes and covariates. Not possible to (easily) model DIF^a^
BCH	Most robust for continuous and count outcomes. Negative weights can be counterintuitive	Recommended	Currently not possible to extend to complex models, or to model DIF
Two‐step approach	Not recommended with continuous or count outcome. Recommended with categorical outcome	Recommended	Recommended, most flexible stepwise approach. Flexible for modelling DIF
LTB	Recommended if no heteroscedasticity is present for continuous and count outcome. Recommended for categorical outcome	Not appropriate	Not appropriate
Classical three‐step	Not recommended	Not recommended	Not recommended

Note

^a^ By DIF we mean differential item functioning, also known as direct effect between the covariate and (some) indicator(s).

A summary of the labelling of these methods in Latent GOLD and Mplus is given in Table 2, and some examples of their use in the Appendix A. The Mplus syntax language further differentiates between ‘automatic’ and manual ML and BCH methods. These are not standalone methods, but different programming choices for them. The ‘automated’ options are not recommended, because of their black‐box approach and strict assumptions. The ‘manual’ options for ML and BCH are very flexible, and comparable in functionalities to the modelling possibilities available in the Latent GOLD syntax language.

**Table 2:** Labeling used in the GUI of mainstream software for the different approaches

Tutorial	Mplus ‘manual’	Latent Gold	Key references
BCH	BCH	BCH	Vermunt (2010)
ML	With equal variances: DE3step	ML	Vermunt (2010)
	With unequal variances: DU3step		
	Covariate: R3step		
LTB	Categorical: Dcat	Only from syntax	Lanza et al. (2013)
	Continuous Dcont		
Two‐step	Only from syntax	Bakk–Kuha in LG 5.1^a^	Bakk and Kuha (2018)

Note

^a^ In earlier versions of Latent GOLD manual implementation is possible. In Appendix A we show the manual version. This can also be implemented in any other software for LC analysis that allows for fixed effect parameters.

### 3.1 Models with covariates

The one‐, two‐ and three‐step methods can all be used when the structural model involves only covariates $Z_{p}$ and one latent class variable. When all of the underlying model assumptions are met (i.e., there are no direct effects between the covariates and the indicators, and all the association between the indicators is explained by the latent variable) and the LC measurement model is strong enough (i.e., entropy $R^{2}$ is above .50), all of these methods give consistent estimates of the parameters of the structural models. The one‐step estimates are then generally the most efficient (i.e., have the smallest standard errors), followed by the two‐step and three‐step estimates, but the differences in efficiency between all of them tend to be small (for such comparisons, see Bakk & Kuha, 2018, and references cited there).

However, the methods differ crucially in how they select and estimate the measurement model which defines the latent classes. In the stepwise approaches this is done in the first step, excluding information about the covariates, and then fixed. In the one‐step approach, in contrast, the measurement model is estimated together with the structural model, and re‐estimated every time the structural model changes. The two estimated models then affect each other, so that every time a new covariate is added or removed the measurement model will change, and even the number of the latent classes suggested by goodness‐of‐fit statistics can change. To avoid the latter problem, the class enumeration at least should be done in a stepwise manner, so that the number of latent classes is selected using only the basic LC model and this number is then fixed when external variables are introduced (Nylund‐Gibson et al., 2019). Even then, the class‐specific measurement probabilities will change when the structural model is changed, thus in effect altering the definition of the latent classes. If these changes are small this is not a major problem. But if the changes in the measurement model are big enough and the latent classes become so distorted that they cannot be meaningfully compared between models with different choices of covariates (for an example, see Bakk & Kuha, 2018). Whether this will happen is only known when we actually fit the models. To avoid these challenges the use of stepwise approaches instead of the one‐step approach is recommended.

In simulation studies where we know that the structural model is correctly specified, the situation where the different estimators differ most is one where the measurement model is weak and the latent classes are poorly separated (the entropy is below .50, say). Estimates from the stepwise approaches can then be biased and will have higher variance than one‐step estimates. The reason for this difference is that the one‐step approach benefits from the extra information in the covariates, which contributes to defining a stronger measurement model (Vermunt, 2010) – the same applies to models where this extra information comes from a distal outcome $Z_{o}$. This situation of a weak measurement model (entropy below .50) is one where the one‐step approach is most recommended, but the analyst should be aware that the interpretation of their latent classes is then particularly strongly driven by the covariates or distal outcomes, and not just the variables Y which are intended as the indicators of the classes.

Another, smaller contrast between the methods arises when we would want to use a different set of observations to estimate the measurement model than the structural model. This may be the case if there is missing data in the covariates, or if we would like to use a completely separate data set to estimate the measurement model (this was the original motivation of two‐step modelling in Xue & Bandeen‐Roche, 2002).

Accommodating this is difficult in one‐step estimation without something like multiple imputation of the missing data, but it is straightforward in stepwise methods. For the estimated conditional probabilities to be transferable from the data set used for the first step to the one used to estimate the structural model the assumption needs to be made that the same measurement model holds in both data sets. Furthermore for three‐step methods we also assume that the distribution of the latent classes is the same in the two data sets (since (6) and (8) depend also on P(X=t)).

### 3.2 Models with distal outcomes

All of the methods considered here again work well for models with a distal outcome $Z_{o}$, if the model assumptions are satisfied. If they are not, however, differences between different methods are larger than they were for models with covariates. Distal outcome models have received a lot of attention in recent years, with several simulation studies looking into their properties under different types of violations of underlying model assumptions (Asparouhov & Muthèn, 2014a, 2014b; Bakk & Vermunt, 2016; Lythgoe et al., 2019; Shin, No, & Hong, 2019; Zhu, Steele, & Moustaki, 2017). Nylund‐Gibson et al. (2019) give a good summary of the existing literature and recommendations, upon which we expand here.

One‐step estimation again suffers from a circularity problem, which is now very obvious (although the problem is ultimately the same even in models with covariates). This is because the outcome $Z_{o}$ that the latent class variable X is supposed to predict in the structural model acts also as another indicator variable in the measurement model, which contributes to the definition of X. It is difficult or impossible to separate these two interpretations, and mainly for this reason we cannot recommend one‐step estimation for distal outcome models.

When the structural model involves continuous or count distal outcomes, even stepwise methods can be sensitive to violations of the distributional assumptions about them. In particular, suppose that $Z_{o}$ given X is taken to be normally distributed with a constant conditional variance. The one‐step, two‐step and ML three‐step estimation make full use of this assumption, and they can thus be biased when the assumptions are violated by non‐normality (skewness or kurtosis) and/or heteroscedasticity of variance within the latent classes (Asparouhov & Muthèn, 2014a, 2014b; Bakk & Kuha, 2018; Bakk & Vermunt, 2016; Shin et al., 2019). For example, Dziak, Bray, Zhang, Zhang, and Lanza (2016) found ML performing almost as badly as one‐step estimation or point estimation in the presence of misspecified numerical distal outcomes, and even worse than it in terms of confidence interval coverage. In essence, the estimation method then has to distort the estimated probabilities of the latent classes and the regression model for distal outcome in order to fit the wrongly assumed conditional distribution. An exception to this is the three‐step BCH approach. Because for continuous outcomes its third step involves (weighted) estimation of a linear regression (ANOVA) model, it avoids (especially when used with robust standard errors) normality assumptions and is insensitive to heteroscedasticity. So from a robustness point of view, BCH can be recommended for models with a continuous distal outcome.

The assumptions about the distribution of a distal outcome are, however, empirically examinable, even with a preliminary naive three‐step method (i.e., by examining models for $Z_{o}$ given assigned class W).^1^ If this suggests problems, they may be reduced by transforming the outcome or by expanding the model to allow for unequal conditional variances for $Z_{o}$ in different latent classes. This should often make two‐step and three‐step ML methods appropriate also in this case. For models with unequal conditional variances the BCH method should be used with modelling the variances as equal to avoid modelling negative variances in some latent classes. If the difference in variances is of interest then the BCH method should be avoided, and ML used instead. Finally, concerns about distributional assumptions are less relevant for models for categorical outcome variables, and any of the stepwise methods can be used for them. However, since BCH can lead to negative frequencies, ML should be preferred.

As noted above, the LTB variant of the one‐step approach (Lanza et al., 2013) was developed specifically to avoid distributional assumptions about a continuous $Z_{o}$. It has also been examined under different conditions by as Asparouhov and Muthèn (2014a) and Asparouhov and Muthèn (2014b) and Bakk, Oberski, and Vermunt (2016). They concluded that while the method works otherwise, it can still yield biased estimates when the variances of $Z_{o}$ are unequal across the latent classes X. This would translate into quadratic effects of $Z_{o}$ in the (multinomial) logistic model for X given $Z_{o}$, and the estimates can be biased if such terms are not included. The LTB method also has the property of all one‐step methods that the definition of the latent classes is affected by the outcome variable.

### 3.3 More complex models

We have so far discussed models which involve a single latent class variable and which include external variables in the form of either covariates only or a single distal outcome only. Both of these limitations can be relaxed, to arrive at models which involve multiple LC variables and/or external variables in multiple roles. The considerations between one‐step and stepwise approaches then remain unchanged, in that one‐step estimation unavoidably leaves the measurement and structural models confused with each other, while stepwise estimation avoids this.

Among the stepwise approaches, the three‐step BCH method becomes unwieldy or unusable when the models get more complex, so it cannot be recommended for such situations (Bray & Dziak, 2018). The ML and two‐step approaches, on the other hand, can still be used essentially unchanged. This is because in their last step they both maximize a log‐likelihood which is of the same form as the one which would be used for one‐step estimation, except with a measurement component (for W in ML, for Y in two‐step estimation) fixed at known values. They can thus be used whenever one‐step estimation would also be feasible, in principle. It should be noted, however, that we have so far very little experience with such more complex models, either in practice or with simulations. They are also not fully implemented in standard software, so some hand‐coding (of the syntax of Mplus or Latent GOLD) would typically be required (e.g., models with two latent variables cannot be run from the GUI of Latent GOLD, but can be easily implemented with its syntax). Similarly, the fully correct standard errors of the estimators are not yet implemented, so that the most accessible options are to ignore the uncertainty from the first step of estimation or to carry out (hand‐coded) bootstrap variance estimation. Similar practical caveats apply also to even more complex models such as multilevel or latent transition models, even though the two‐step and ML approaches are generalizable to them as well (Bray & Dziak, 2018; Di Mari & Bakk, 2018).

Another kind of extension is to models where the measurement model is generalized to allow for direct associations between external variables and indicators $Y_{k}$, even conditional on latent class X. This would be the case, in particular, if we wanted to consider such direct paths between covariates $Z_{p}$ and the indicators, to examine or allow for non‐invariance of measurement (differential item functioning) with respect to the covariates. Such direct effects can be accommodated, with some additional complexity, in two‐step (Di Mari & Bakk, 2018) and three‐step estimation (Vermunt & Magidson, 2020). Some examinations of this situation have recently been carried out by Di Mari and Bakk (2018) and Janssen, van Laar, de Rooij, Kuha, and Bakk (2019), but the performance of the different estimators in different circumstances here is still a question for further research.

In conclusion, based on the available data for complex LC models the two‐step estimator seems to have the most desirable properties: flexibility of modelling (a quality lacking from the three step approaches) and good estimation speed (which improves compared to the one‐step approach since the same parameters do not need to be re‐estimated after every modification in the model).

## 4 Example applications

In the following we illustrate the use of the different estimators via two real data examples using data from the 1976–77 General Social Survey, a cross‐sectional survey conducted in the USA by the National Opinion Research Center (1977). The first example illustrates a model with a continuous distal outcome predicted by a single LC variable. The second example includes two LC variables, one as the outcome and the other as one of the covariates.

### 4.1 A distal outcome model: predicting income from social status

Here we consider a model which includes a person's social status, specified as an LC variable, as a predictor for the person's income. The indicators for social status are the respondent's father's and mother's education and the prestige of the father's occupation. Education is measured in five categories, ranging from ‘lower than high school’ to ‘graduate’. The father's job prestige is measured on a scale from 12 to 82 that we have recoded into three categories: low (up to 36), medium (37–61) and high (62 or above). This recoding and the initial LC analysis are described in more detail in Bakk et al. (2016). The best‐fitting measurement model has three latent classes, as shown in Table 3. We label them ‘low’, ‘medium’ and ‘high’ social class, with estimated proportions of 69%, 24% and 7% of the respondents, respectively. We then related this social status variable to the respondent's real income (measured in thousands of dollars) as a distal outcome.

**Table 3:** The latent class model for social class

Social status	Low	Medium	High
Size	.69	.24	.07
Father's job status			
Low	.47	.31	.05
Medium	.53	.67	.46
High	.00	.02	.49
Mother's education			
Below high school	.83	.14	.15
High school	.16	.78	.44
Junior college	.00	.03	.01
Bachelor	.01	.04	.30
Graduate	.00	.01	.10
Father's education			
Below high school	.95	.08	.01
High school	.05	.86	.12
Junior college	.00	.00	.05
Bachelor	.00	.05	.38
Graduate	.00	.00	.43

Results from different ways of estimating this model are shown in Table 4. The substantive conclusion using all approaches is that income is highest among the group with the highest social status at birth, and lowest among the lowest, suggesting persistence of social position between the respondent's and their parents' generations. The estimated magnitudes of the income differences between the latent classes are, however, rather different between the different modelling approaches. Knowing that the BCH approach is the most robust for models with continuous distal outcomes, we can compare its results with the other estimates. For the one‐step estimates this comparison is not really possible, because the latent classes themselves get distorted from the ones in Table 3 when the income variable is added to the model (thus these estimates are not reported here). Among the stepwise methods, all but the LTB estimates show a stronger association between social status and mean income than is indicated by the BCH estimates. The reason for this overestimation is most likely that the distribution of income is skewed and has a variance which increases across the social status classes (see the last row of Table 4, which shows its class‐specific variances). Two‐step and ML estimates which allow for unequal conditional variances are also shown in Table 4, but they do not fully remove the difference to the BCH estimates. A still closer agreement between the different estimates could be obtained by considering log‐income as the distal outcome, to reduce its skewness.

**Table 4:** Predicting income from social class with the different approaches

Method	Conditional			
	variances^a^	$\mu_{1}$ (low)	$\mu_{2}$ (medium)	$\mu_{3}$ (high)
BCH		26.65 (0.60)	37.06 (1.35)	44.70 (3.09)
ML	Equal	25.59 (0.50)	33.22 (2.33)	113.21 (23.59)
ML	Unequal	20.63 (0.31)	44.94 (1.06)	65.67 (3.84)
LTB		25.37 (0.90)	36.49 (1.16)	44.16 (2.62)
Two‐step	Equal	26.90 (0.58)	34.86 (1.77)	51.04 (6.48)
Two‐step	Unequal	24.72 (0.67)	36.54 (1.49)	64.03 (3.78)
One‐step		LC model distorted		
Variances BCH		538.43 (47.68)	713.64 (104.39)	1,209.44 (252.19)

Note

^a^ The standard errors of BCH estimates are calculated assuming unequal conditional variances. The one‐step estimates are distorted under both equal and unequal conditional variances.

### 4.2 A model with two latent‐class variables: predicting tolerance towards minorities from social status, education and age

Here we add a second LC variable. It describes the respondent's level and pattern of tolerance towards different minorities, based on five questions which refer separately to tolerance towards communists, militarists, racists, atheists and homosexuals. Latent class analysis of these items was originally carried out by McCutcheon (1985) and replicated by Bakk et al. (2014), and more information on the data and the analysis can be found there.^2^ A four‐class model clusters respondents into the intolerant (57% of the respondents), those who are tolerant towards all minorities (21%), and two classes which we label ‘intolerant of left’ (11%) and ‘intolerant of right’ (11%), as shown in Table 5.

**Table 5:** The tolerance latent classes

	Tolerant	Intolerant	Intolerant of left	Intolerant of right
Size	.21	.57	.11	.11
Tolerance for				
Racists	.90	.08	.78	.02
Communists	.94	.04	.25	.62
Militarists	.91	.05	.39	.36
Atheists	.98	.03	.62	.41
Homosexuals	.96	.14	.55	.73

We regress these tolerance classes on the respondent's social status, education and birth cohort. Based on the analysis performed by McCutcheon, education was recoded into three categories (lower than high school, high school, beyond high school), and birth cohort was coded into four categories (‘old’, born before 1914; ‘middle’ aged, born in 1915–1933; ‘young middle’ aged, born in 1934–1951; and ‘young’, born after 1951). Social status was included in the form of the LC variable defined in the first example. This second example thus illustrates a model where an LC variable depends on covariates and where, further, one of the covariates is itself latent (the fitted model also includes a regression model for social status given education and birth cohort, to allow for associations among the covariates).

This type of analysis can only be performed using the one‐step, two‐step and ML approaches. The LTB approach is not applicable when the latent class is the outcome variable, and the BCH approach cannot handle more than a single latent variable. As in the first example, here one‐step estimation again results in an estimated model where the definition of the tolerance classes has changed substantially, and we do not report these results. Table 6 thus reports the estimated coefficients in the structural model for the tolerance classes, obtained using the three‐step ML approach and the two‐step approach. The substantive conclusions are very similar between them. We can see that controlling for education and cohort, people with low social status have a lower probability of being tolerant, while people with higher status tend to be more tolerant. People of higher social status are also more intolerant of the left than the other groups. Controlling for the other variables, people with higher education tend to be more tolerant, and less intolerant or intolerant of the left. Older people tend to be more intolerant and younger people more tolerant.

**Table 6:** Predicting tolerance with the ML and two step approaches

	Tolerant	Intolerant	Intolerant of right	Intolerant of left
ML				
Social status (latent)				
Low	−.48 (.11)	.33 (.11)	−.16 (.18)	.31 (.23)
Medium	.05 (.12)	−.18 (.13)	.07 (.19)	.06 (.27)
High	.43 (.17)	−.15 (.18)	.09 (.28)	−.37 (.41)
Education				
Lower	−.35 (.11)	.36 (.07)	−.25 (.15)	.24 (.14)
High school	−.22 (.08)	−.04 (.06)	.25 (.11)	.01 (.11)
Higher	.57 (.08)	−.32 (.08)	.00 (.14)	−.25 (.14)
Cohort				
Young	.39 (.1017)	−.84 (.10)	.44 (.14)	.01 (.15)
Young‐middle	.39 (.09)	−.23 (.07)	−.13 (.13)	−.03 (.13)
Middle	−.11 (.11)	.32 (.07)	−.08 (.15)	−.13 (.16)
Old	−.67 (.17)	.75 (.09)	−.22 (.23)	.15 (.19)
Two step				
Social status (latent)				
Low	−.42 (.16)	.29 (.17)	−.25 (.22)	.38 (.40)
Medium	.01 (.15)	−.36 (.18)	.04 (.21)	.31 (.39)
High	.41 (.28)	.06 (.33)	.21 (.39)	−.69 (.76)
Education				
Lower	−.48 (.10)	.40 (.06)	−.30 (.13)	.38 (.11)
High school	−.18 (.07)	−.02 (.06)	.28 (.10)	−.08 (.10)
Higher	.66 (.07)	−.38 (.07)	.02 (.11)	−.30 (.11)
Cohort				
Young	.40 (.09)	−.81 (.09)	.35 (.13)	.06 (.14)
Young‐middle	.33 (.08)	−.25 (.06)	−.10 (.11)	.02 (.11)
Middle	−.18 (.10)	.31 (.07)	−.09 (.13)	−.04 (.13)
Old	−.54 (.14)	.75 (.08)	−.16 (.18)	−.05 (.16)

## 5 Discussion

In this paper we have provided an overview of currently existing approaches for relating latent class membership to external variables, and given practical recommendations for applied researchers about which of the multitude of approaches is best suited to different modelling situations. We compared one‐step estimation of the LC model with the newly developed bias‐adjusted (ML and BCH) three‐step (Vermunt, 2010), two‐step (Bakk & Kuha, 2018), and LTB (Lanza et al., 2013) approaches.

The crucial disadvantage of the one‐step approach is that the LC measurement model is re‐evaluated every time the structural model is changed, which can mean that the definition of the latent classes will also keep changing. The stepwise three‐step and two‐step methods eliminate this problem. All of them work roughly equally well for models with external covariates. For models with distal outcomes, the BCH method is the safest approach, because it is the most robust approach against misspecifications of the class‐specific distribution of the outcome. However, with due care the other stepwise methods can also be used in this situation. This means that one should carefully monitor whether the underlying assumptions are met, and interpret the results with caution if the key assumptions are violated.

Our experience with other modelling situations is still limited and evolving. With more complex structural models (e.g., multilevel LC models or Markov models) the BCH method soon becomes difficult to adopt, but the ML and two‐step methods extend to them easily. For generalising the measurement model, the stepwise methods can accommodate models with direct covariate effects to capture differential item functioning, but more research is needed to better understand their performance in that situation.

We demonstrated and compared the different methods using models for data from the US General Social Survey. We first considered models for predicting income from social status of parents represented by three latent classes. The direction of the substantive conclusion was the same from all of the methods, but the sizes of the associations varied widely, and with the one‐step method the definition of the latent classes was also unstable. We then demonstrated a model with two LC variables, for predicting tolerance towards minorities (in four latent classes) from (latent class) social status and observed education. The Latent GOLD syntax for these examples is included in the Appendix A.

The two real data examples have highlighted the weakness of the one‐step approach that we have discussed above, namely that when the model complexity increases the LC variable will be re‐estimated and may change substantially, and the new classes cannot be meaningfully compared to the simple measurement model. Furthermore, the overall fit statistics can also show large misfits, and it is hard to identify which part of the model they are coming from. All these issues do not represent a problem for any of the stepwise estimators. Furthermore, in the second example we showed the ease with which the ML and two‐step approaches can be extended to models with multiple latent variables, a strength that does not characterize the BCH approach. Nevertheless, the first example showed that all methods except the BCH are sensitive to distributional assumptions of the distal outcome, and while the main conclusions did not change while using the ML and two‐step approaches in this example, the differences in the magnitude of the parameters were substantial.

While creating this overview, some recommendations for future research arose from our literature search. There is a gap in the literature on stepwise LC modelling for more complex models, with multiple latent class variables, multilevel LC models, or for longitudinal models such as latent Markov or latent growth models. Only a few articles present stepwise estimators for these situations, and there is no overarching simulation study that evaluates the different estimators for them. Future research should also investigate the issue of model selection and class enumeration for complex models.

## Conflicts of interest

All authors declare no conflict of interest.

## Data Availability

The data that support the findings of this study are openly available in the data repository of the National Opinion Research, General Social Survey at http://doi.org/10.3886/ICPSR07573.v1.

## Footnotes

^1^ In addition, Mplus offers some ‘safety checks' for the ML approach: it reports if a large switch in class probabilities took place, and does not provide the step 3 estimates.

^2^ While the original analysis was performed using listwise deletion, we included all the missing data to be in line with the previous model and to show the flexibility of the stepwise approaches in this sense. As a consequence the LC model parameters are somewhat different than in the papers cited.

## Appendix A

### Latent GOLD example syntax for the different modelling approaches

#### Stepwise approaches in Latent GOLD

When using a bias‐adjusted step‐three approaches (ML and BCH) in Latent GOLD, one first estimates an LC model and saves the classification information and other variables of interest to an output file. In the third step, this output file is used as the data set to be analyzed. The class assignments are related to the distal outcome as follows:

**Table:**

options step3 modal ml; output parameters = effect standarderrors = robust; variables dependent inc_1000 continuous; latent Cluster nominal posterior = (clusterstatus#1 clusterstatus#2 clusterstatus#3); equations inc_1000 <‐ 1 + Cluster; inc_1000|cluster;

The choice of the type of three‐step approach is defined by the command line ‘step3 << modal/proportional ml/ bch>> simultaneous’. In the definition of the LC variable one specifies the variables in the data file containing the posterior classification probabilities from the first step: ‘(Cluster #1 Cluster #2 Cluster #3)’.

### Using the LTB approach in Latent GOLD

**Table:**

output parameters = effect betaopts = wl standarderrors = npbootstrap profile = LTB classification classification = model estimatedvalues = model; variables dependent paprecat nominal, madeg nominal, padeg nominal; independent inc_1000; latent Cluster nominal 3; equations Cluster <‐ 1 + inc_1000; paprecat <‐ 1 + Cluster; madeg <‐ 1 + Cluster; padeg <‐ 1 + Cluster;

The profile = LTB option will provide the class‐specific means and the corresponding standard errors. In current syntax the standard error is estimated using nonparametric bootstrap, the jackknife, and standard options can also be chosen.

### Two‐step model predicting income from social status

As of version 6.0 of Latent GOLD, by selecting the Bakk–Kuha method from the GUI one can run simple two‐step methods. For more complex models syntax can be used using the option latent Cluster nominal posterior = ( logdensity#1 logdensity#2 logdensity#3);. The fixed values can also be read in from a text file. Moreover, the text file can also contain their covariance matrix. The covariance matrix is then used to correct the standard errors of the step 2 parameters for uncertainty in the step 1 parameters (a step that we skipped for simplicity here). We present below the syntax option that can be run also in earlier versions of Latent GOLD.

**Table:**

output parameters = effect betaopts = wl standarderrors = robust profile probmeans = posterior estimatedvalues = model; variables dependent paprecat nominal, madeg nominal, padeg nominal, inc_1000 continuous; independent education nominal, cohort_ nominal; latent Clusterstatus nominal 3; equations clusterstatus <‐(a1)1; paprecat <‐ (k1)1 + (k2)clusterstatus; madeg <‐ (k3)1 + (k4)clusterstatus; padeg <‐ (k5)1 + (k6)clusterstatus; inc_1000 <‐ 1 + clusterstatus; inc_1000; a1 k1 k2 k3 k4 k5 k6 ={<<fixed values>>}

Using the two‐step approach the syntax, contains the full measurement and structural model in the second step, keeping the parameters of the measurement model fixed.

#### Two step model with two latent variables: predicting tolerance from social status and education

**Table:**

output parameters = effect betaopts = wl standarderrors = robust profile probmeans = posterior estimatedvalues = model; variables dependent racist_ nominal, communists_ nominal, militarists_ nominal, atheists_ nominal, homtest nominal, paprecat nominal, madeg nominal, padeg nominal; independent education nominal, cohort_ nominal; latent ClusterTol nominal 4, clusterstatus nominal 3; equations clusterTol <‐ 1 + clusterstatus+ education + cohort_; clusterstatus <‐(a1)1 + education; homtest <‐ (a)1 + (b)ClusterTol; racist_ <‐ (c)1 + (d)ClusterTol; communists_ <‐ (e)1 + (f)ClusterTol; militarists_ <‐ (g)1 + (h)ClusterTol; atheists_ <‐ (i)1 + (j)ClusterTol; paprecat <‐ (k1)1 + (k2)clusterstatus; madeg <‐ (k3)1 + (k4)clusterstatus; padeg <‐ (k5)1 + (k6)clusterstatus; a b c d e f g h i j k1 k2 k3 k4 k5 k6 ={<<fixed values>>}

### ML approach with two latent variables: predicting tolerance from social status and education

**Table:**

options step3 modal ml; output parameters = effect standarderrors = robust; variables independent education nominal, cohort_ nominal; latent ClustersocialStatus nominal posterior = ( clustersocialstatus#1 clustersocialstatus#2 clustersocialstatus#3), ClusterTolerance nominal posterior = ( clusterTolerance#1 clusterTolerance#2 clusterTolerance#3 clusterTolerance#4); equations clusterTolerance <‐ 1 + ClustersocialStatus +Education + cohort_; ClustersocialStatus <‐ 1 + Education;

## References

[bmsp12227-bib-0001] Asparouhov, T. , & Muthèn, B. (2014a). Auxiliary variables in mixture modeling: Three‐step approaches using mplus. Structural Equation Modeling: A Multidisciplinary Journal, 21, 329–341. 10.1080/10705511.2014.915181

[bmsp12227-bib-0002] Asparouhov, T. , & Muthén, B. (2014b). Auxiliary variables in mixture modeling: Using the bch method in Mplus to estimate a distal outcome model on an arbitrary secondary model. Retrieved from http://www.statmodel.com/examples/webnotes/webnote21.pdf

[bmsp12227-bib-0003] Bakk, Z. , & Kuha, J. (2018). Two‐step estimation of models between latent classes and external variables. Psychometrika, 83, 871–892. 10.1007/s11336-017-9592-7 29150817

[bmsp12227-bib-0004] Bakk, Z. , Oberski, D. , & Vermunt, J. (2014). Relating latent class assignments to external variables: Standard errors for correct inference. Political Analysis, 22, 520–540. 10.1093/pan/mpu003

[bmsp12227-bib-0005] Bakk, Z. , Oberski, D. L. , & Vermunt, J. K. (2016). Relating latent class membership to continuous distal outcomes: Improving the LTB approach and a modified three‐step implementation. Structural Equation Modeling: A Multidisciplinary Journal, 23, 278–289. 10.1080/10705511.2015.1049698

[bmsp12227-bib-0006] Bakk, Z. , Tekle, F. T. , & Vermunt, J. K. (2013). Estimating the association between latent class membership and external variables using bias‐adjusted three‐step approaches. Sociological Methodology, 43, 272–311. 10.1177/0081175012470644

[bmsp12227-bib-0007] Bakk, Z. , & Vermunt, J. K. (2016). Robustness of stepwise latent class modeling with continuous distal outcomes. Structural Equation Modeling: A Multidisciplinary Journal, 23, 20–31. 10.1080/10705511.2014.955104

[bmsp12227-bib-0008] Bandeen‐Roche, K. , Miglioretti, D. L. , Zeger, S. L. , & Rathouz, P. J. (1997). Latent variable regression for multiple discrete outcomes. Journal of the American Statistical Association, 92, 1375–1386.

[bmsp12227-bib-0009] Bolck, A. , Croon, M. , & Hagenaars, J. (2004). Estimating latent structure models with categorical variables: One‐step versus three‐step estimators. Political Analysis, 12, 3–27.

[bmsp12227-bib-0010] Bray, B. C. , & Dziak, J. J. (2018). Commentary on latent class, latent profile, and latent transition analysis for characterizing individual differences in learning. Learning and Individual Differences, 66, 105–110. 10.1016/j.lindif.2018.06.001 (Modelling individual differences in students' cognitions and development: Latent variable mixture model approaches).

[bmsp12227-bib-0011] Bray, B. C. , Lanza, S. T. , & Tan, X. (2015). Eliminating Bias in Classify‐Analyze Approaches for Latent Class Analysis. Structural Equation Modeling: A Multidisciplinary Journal, 22, 1–11. 10.1080/10705511.2014.935265 (PMID: 25614730) 25614730 PMC4299667

[bmsp12227-bib-0012] Di Mari, R. , & Bakk, Z. (2018). Mostly harmless direct effects: A comparison of different latent Markov modeling approaches. Structural Equation Modeling: A Multidisciplinary Journal, 25, 467–483. 10.1080/10705511.2017.1387860

[bmsp12227-bib-0013] Dziak, J. J. , Bray, B. C. , Zhang, J. , Zhang, M. , & Lanza, S. T. (2016). Comparing the performance of improved classify‐analyze approaches for distal outcomes in latent profile analysis. Methodology: European Journal of Research Methods for the Behavioral Social Sciences, 12, 107–116. 10.1027/1614-2241/a000114 28630602 PMC5473653

[bmsp12227-bib-0014] Fagginger Auer, M. F. , Hickendorff, M. , Van Putten, C. M. , Bèguin, A. A. , & Heiser, W. J. (2016). Multilevel latent class analysis for large‐scale educational assessment data: Exploring the relation between the curriculum and students' mathematical strategies. Applied Measurement in Education, 29, 144–159. 10.1080/08957347.2016.1138959

[bmsp12227-bib-0015] Geary, D. C. , Bailey, D. H. , Littlefield, A. , Wood, P. , Hoard, M. K. , & Nugent, L. (2009). First‐grade predictors of mathematical learning disability: A latent class trajectory analysis. Cognitive Development, 24, 411–429. 10.1016/j.cogdev.2009.10.001 (Atypical Development of Numerical Cognition). PMC2813681 20046817

[bmsp12227-bib-0016] Gong, G. , & Samaniego, F. J. (1981). Pseudo maximum likelihood estimation: Theory and applications. The Annals of Statistics, 9, 861–869. 10.1214/aos/1176345526

[bmsp12227-bib-0017] Goodman, L. A. (1974). The analysis of systems of qualitative variables when some of the variables are unobservable. Part I: A modified latent structure approach. American Journal of Sociology, 79–259. 10.1086/225676

[bmsp12227-bib-0018] Hagenaars, J. A. (1990). Categorical longitudinal data‐loglinear analysis of panel, trend and cohort data. Newbury Park, CA: Sage.

[bmsp12227-bib-0019] Janssen, J. H. M. , van Laar, S. , de Rooij, M. J. , Kuha, J. , & Bakk, Z. (2019). The detection and modeling of direct effects in latent class analysis. Structural Equation Modeling: A Multidisciplinary Journal, 26, 280–290. 10.1080/10705511.2018.1541745

[bmsp12227-bib-0020] Lanza, T. S. , Tan, X. , & Bray, C. B. (2013). Latent class analysis with distal outcomes: A flexible model‐based approach. Structural Equation Modeling, 20, 1–26. 10.1080/10705511.2013.742377 25419096 PMC4240499

[bmsp12227-bib-0021] Lythgoe, D. T. , Garcia‐Fiñana, M. , & Cox, T. F. (2019). Latent class modeling with a time‐to‐event distal outcome: A comparison of one, two and three‐step approaches. Structural Equation Modeling: A Multidisciplinary Journal, 26, 51–65. 10.1080/10705511.2018.1495081

[bmsp12227-bib-0022] Magidson, J. (1981). Qualitative variance, entropy, and correlation ratios for nominal dependent variables. Social Science Research, 10, 177–194. 10.1016/0049-089X(81)90003-X

[bmsp12227-bib-0023] McCutcheon, A. L. (1985). A latent class analysis of tolerance for nonconformity in the American public. Public Opinion Quarterly, 49, 474–488. 10.1086/268945

[bmsp12227-bib-0024] McCutcheon, A. L. (1987). Latent class analysis. Newbury Park, CA: Sage.

[bmsp12227-bib-0025] Mulder, E. , Vermunt, J. , Brand, E. , Bullens, R. , & Van Merle, H. (2012). Recidivism in subgroups of serious juvenile offenders: Different profiles, different risks? Criminal Behaviour and Mental Health, 22, 122–135. 10.1002/cbm.1819 22213477

[bmsp12227-bib-0026] Mustillo, T. J. (2009). Modeling new party performance: A conceptual and methodological approach for volatile party systems. Political Analysis, 17, 311–332. 10.1093/pan/mpp007

[bmsp12227-bib-0027] Muthén, L. K. , & Muthén, B. O. (2017). Mplus user's guide (8th ed.) [Computer software manual]. Los Angeles, CA: Muthén & Muthén.

[bmsp12227-bib-0028] National Opinion Research Center (1977). General social survey 1976–1977. Boston, MA: National Opinion Research Center. 10.3886/ICPSR07398.v1

[bmsp12227-bib-0029] Nylund‐Gibson, K. , Grimm, R. P. , & Masyn, K. E. (2019). Prediction from latent classes: A demonstration of different approaches to include distal outcomes in mixture models. Structural Equation Modeling: A Multidisciplinary Journal, 26, 967–985. 10.1080/10705511.2019.1590146

[bmsp12227-bib-0030] Petras, H. , & Masyn, K. (2010). General growth mixture analysis with antecedents and consequences of change. In A. Piquero & D. Weisburd (Eds.), Handbook of quantitative criminology (pp. 69–100). New York, NY: Springer.

[bmsp12227-bib-0031] Shin, M. , No, U. , & Hong, S. (2019). Comparing the robustness of stepwise mixture modeling with continuous nonnormal distal outcomes. Educational and Psychological Measurement, 79, 1156–1183. 10.1177/0013164419839770 31619843 PMC6777068

[bmsp12227-bib-0032] Vermunt, J. K. (2010). Latent class modeling with covariates: Two improved three‐step approaches. Political Analysis, 18, 450–469. 10.1093/pan/mpq025

[bmsp12227-bib-0033] Vermunt, J. K. , & Magidson, J. (2005). Latent GOLD 4.0 user's guide. Belmont, MA: Statistical Innovations.

[bmsp12227-bib-0034] Vermunt, J. K. , & Magidson, J. (2016). Technical guide for Latent GOLD 5.1: Basic, advanced and syntax. Belmont, MA: Statistical Innovations.

[bmsp12227-bib-0035] Vermunt, J. K. , & Magidson, J. (2020). How to perform three‐step latent class analysis in the presence of measurement non‐invariance or differential item functioning. Retrieved from https://jeroenvermunt.nl/VermuntMagidson2020.pdf

[bmsp12227-bib-0036] Wedel, M. , ter Hofstede, F. , & Steenkamp, J. (1998). Mixture model analysis of complex samples. Journal of Classification, 15, 225–244. 10.1007/s003579900032

[bmsp12227-bib-0037] Xue, Q.‐L. , & Bandeen‐Roche, K. (2002). Combining complete multivariate outcomes with incomplete covariate information: A latent class approach. Biometrics, 58, 110–120. 10.1111/j.0006-341X.2002.00110.x 11890305

[bmsp12227-bib-0038] Zhu, Y. , Steele, F. , & Moustaki, I. (2017). A general 3‐step maximum likelihood approach to estimate the effects of multiple latent categorical variables on a distal outcome. Structural Equation Modeling: A Multidisciplinary Journal, 24, 643–656. 10.1080/10705511.2017.1324310

